# Mitochondria play an important role in the cell proliferation suppressing activity of berberine

**DOI:** 10.1038/srep41712

**Published:** 2017-02-09

**Authors:** Xiao-Jin Yan, Xuan Yu, Xin-Pei Wang, Jing-Fei Jiang, Zhi-Yi Yuan, Xi Lu, Fan Lei, Dong-Ming Xing

**Affiliations:** 1 MOE Key Laboratory of Protein Sciences, Laboratory of Molecular Pharmacology and Pharmaceutical Sciences, School of Life Sciences, Tsinghua University, Beijing 100084, China

## Abstract

After being studied for approximately a century, berberine (BBR) has been found to act on various targets and pathways. A great challenge in the pharmacological analysis of BBR at present is to identify which target(s) plays a decisive role. In the study described herein, a rescue experiment was designed to show the important role of mitochondria in BBR activity. A toxic dose of BBR was applied to inhibit cell proliferation and mitochondrial activity, then α-ketobutyrate (AKB), an analogue of pyruvate that serves only as an electron receptor of NADH, was proven to partially restore cell proliferation. However, mitochondrial morphology damage and TCA cycle suppression were not recovered by AKB. As the AKB just help to regenerate NAD+, which is make up for part function of mitochondrial, the recovered cell proliferation stands for the contribution of mitochondria to the activity of BBR. Our results also indicate that BBR suppresses tumour growth and reduces energy charge and mitochondrial DNA (mtDNA) copy number in a HepG2 xenograft model. In summary, our study suggests that mitochondria play an important role in BBR activity regarding tumour cell proliferation and metabolism.

Berberine (BBR), a benzylisoquinoline alkaloid, is an active constituent of many medicinal plants^1^. BBR has been characterized in previous studies by its diverse pharmacodynamics^2^,^3^. Such multiple functions imply that BBR may regulate basic bioprocess on cell proliferation and metabolism, for example, BBR is reported to stimulate glycolysis and fatty acid consumption and suppress DNA replication and transcription^4^,^5^,^6^,^7^,^8^. In addition, BBR has been proven to reduce mitochondrial activity^9^.

Corresponding to the multiple functions, BBR has been found to impact on many molecules and cellular pathways. One prevailing hypothesis is that BBR binds to nucleic acids and disturbs DNA and RNA metabolism^4^,^10^. Another representative hypothesis claims that, depending on the transmembrane potential, BBR accumulates on the inner membrane of mitochondria and inhibits mitochondrial respiratory complex I activity^11^,^12^, thus, BBR triggers oxidative stress, mitochondrial swelling and apoptosis^13^,^14^. Moreover, BBR is believed to interplay with various enzymes, receptors and iron channels^15^.

Although great progress on understanding the activity of BBR has been made in previous studies, the role of each target in BBR activity remains unclear. After entering the cell, BBR should bind to and interact with different targets simultaneously; therefore, the activity of BBR represents a summational effect of all of its targets. However, most previous studies have been screening investigations, and each has focused on one of the effects of BBR and reported a correlation or causation between its effect and one of its target. No study has succeeded in measuring the weights of different targets or has determined which target(s) plays a decisive role.

Due to the limitations discussed above, the decisive target of BBR remains unclear. In this study, cell proliferation was chosen as an indicator of the effect of BBR, and a rescue experiment was designed to show the contribution of mitochondria to the integral activity of BBR. The inhibitory effect of BBR on cell proliferation and mitochondrial activity was investigated, and α-ketobutyrate (AKB), an exogenous electron receptor of NADH, was proven to restore cell proliferation but not mitochondrial activity in the study. Moreover, we further investigated the effect of BBR on the regulation of cell metabolism and mitochondrial DNA copy number.

## Results

### Pyruvate restores BBR-induced cell proliferation inhibition

To investigate the role of mitochondria in BBR activity, we were inspired by a study led by Professor Vander Heiden^16^ and designed an experiment to test whether pyruvate is able to rescue cell proliferation inhibited by BBR.

**Figure 1:**
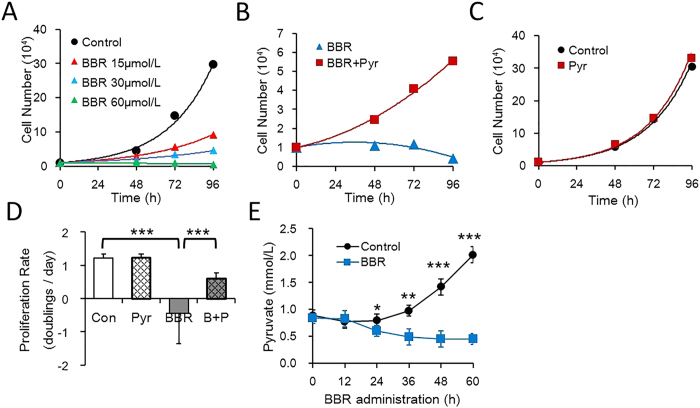
Cell proliferation inhibition by BBR is restored by pyruvate. Cell counts was conducted over time with a hemocytometer, cell numbers were normalized to 1 at t = 0 when media conditions were applied. (**A**) Number of HepG2 cells with BBR gradient treatment. (**B**) Number of HepG2 cells with the indicated treatment. BBR 60 μmol/L, Pyr (Pyruvate) 1 mmol/L. (**C**) Number of HepG2 cells cultured with or without 1 mmol/L Pyruvate. (**D**) Proliferation rate of HepG2 cells with the indicated treatment. Con, control; BBR 60 μmol/L; AKB 1 mmol/L; A + B: BBR 60 μmol/L and AKB 1 mmol/L. Mean ± SEM; ***p < 0.001, n = 3. (**E**) Concentration of pyruvate in the media of HepG2 cells was determined over time by a pyruvate kit. BBR 60 μmol/L. Mean ± SEM; *p < 0.05, **p < 0.01, ***p < 0.001, n = 3.

First, a cell counting assay was conducted to determine the dose of BBR that can completely suppress HepG2 cell proliferation. The results showed that BBR inhibited cell proliferation in a dose-dependent manner and at 60 μmol/L BBR, the cell number was maintained (Fig. 1A). This concentration was thus chosen for subsequent experiments. Next, HepG2 cells were treated with 60 μmol/L BBR in the absence or presence of pyruvate, which partially recovered the proliferation inhibited by BBR (Fig. 1B), but did not significantly change the number of control cells (Fig. 1C). The cell proliferation rate of the pyruvate group was approximately half that of the control cells (Fig. 1D); therefore, pyruvate recovered cell proliferation to almost half of the initial rate. It was also found that cultured HepG2 cells liberated pyruvate into the medium, whilst BBR administration reduced pyruvate concentration relative to the starting point (Fig. 1E), suggesting that BBR increased net consumption of exogenous pyruvate. Thus, BBR caused pyruvate auxotrophy in HepG2 cells. As pyruvate partially restored the cell proliferation inhibited by BBR, pyruvate metabolism may play an important role in BBR activity.

### Pyruvate and α-ketobutyrate serve as alternative electron receptors

**Figure 2:**
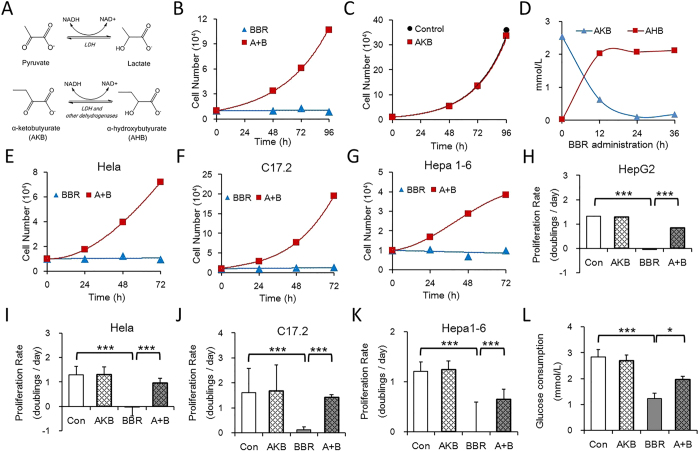
Exogenous Pyruvate or AKB serves as the alternative electron receptors as BBR blocks NAD+ regeneration. (**A**) Pyruvate and AKB are substrates of lactate dehydrogenase (LDH) and other dehydrogenases, which receive electron from NADH and help to regenerate NAD+ (Referred from Sullivan *et al*.^16^). (**B**) Number of HepG2 cells with the indicated treatment. BBR 60 μmol/L, AKB (α-ketobutyrate) 1 mmol/L. (**C**) Number of HepG2 cells cultured with the indicated treatment. (**D**) Concentration of AKB and AHB in the media of HepG2 cells was determined over time by LC-MS/MS analysis. BBR 60 μmol/L. (**E–G**) Number of Hela, C17.2 and HepG2 cells with the indicated treatment. BBR: 60 μmol/L; A + B: BBR 60 μmol/L and AKB 1 mmol/L. (**H**–**K**) Proliferation rate of HepG2, Hela, C17.2 and Hepa1-6 cells with the indicated treatment. Con, control; BBR 60 μmol/L; AKB 1 mmol/L; A + B, BBR 60 μmol/L and AKB 1 mmol/L. Mean ± SEM; ***p < 0.001, n = 3. (**L**) Glucose consumption of HepG2 cells cultured with or without AKB and BBR. Glucose consumption is defined as the difference between the initial glucose concentration and the terminal glucose concentration in the medium. Con, control; BBR 60 μmol/L; AKB 1 mmol/L; A + B, BBR 60 μmol/L and AKB 1 mmol/L. Mean ± SEM; *p < 0.05, ***p < 0.001, n = 3.

Pyruvate not only provides cell carbon substrate for biosynthesis but also serves as an electron acceptor^16^,^17^. Thus, it was still uncertain as to which process accounts for the recovered cell proliferation caused by a toxic dose of BBR. To reduce the number of possibilities, AKB was utilized to replace pyruvate. AKB is an exogenous analogue of pyruvate that receives an electron from NADH in lactate dehydrogenase (LDH) and other dehydrogenase-catalysed reactions and facilitates NAD+ regeneration (Fig. 2A) but does not provide cell carbon^16^.

The results showed that AKB was transformed into α-hydrobutyrate (AHB) (Fig. 2D) and partially recovered BBR induced proliferation stagnation in HepG2 cells (Fig. 2B), whereas it did not significantly influence cell proliferation in control cells (Fig. 2C). In addition, this cell proliferation inhibition/recovery experiment can be used in other tumour cells from different tissues and species (Fig. 2E–G), representing a universal application. Therefore, AKB could serve as a substitute for pyruvate to rescue the cell proliferation inhibited by BBR. Glucose provides cell energy and carbon via glycolysis and the TCA cycle, and a high level of glycolysis is essential for tumour cell proliferation^18^. Consistent with the observed cell proliferation inhibition, BBR reduced glucose consumption and AKB improved glucose consumption in HepG2 cells (Fig. 2L), which may have contributed to the recovered cell proliferation. In summary, these results suggest that BBR inhibits cell proliferation and glucose metabolism and that the exogenous electron acceptors pyruvate and AKB can revert this inhibition.

### BBR damages the mitochondrial structure and AKB does not recover that damage

Previous studies have reported that BBR suppresses mitochondrial activity and leads to mitochondrial swelling^9^,^12^,^13^. As AKB rescued cell proliferation and glucose consumption reduced by BBR, we next investigated whether BBR suppresses cell proliferation via inhibiting mitochondrial activity and whether AKB is able to revert BBR-reduced mitochondrial activity.

**Figure 3:**
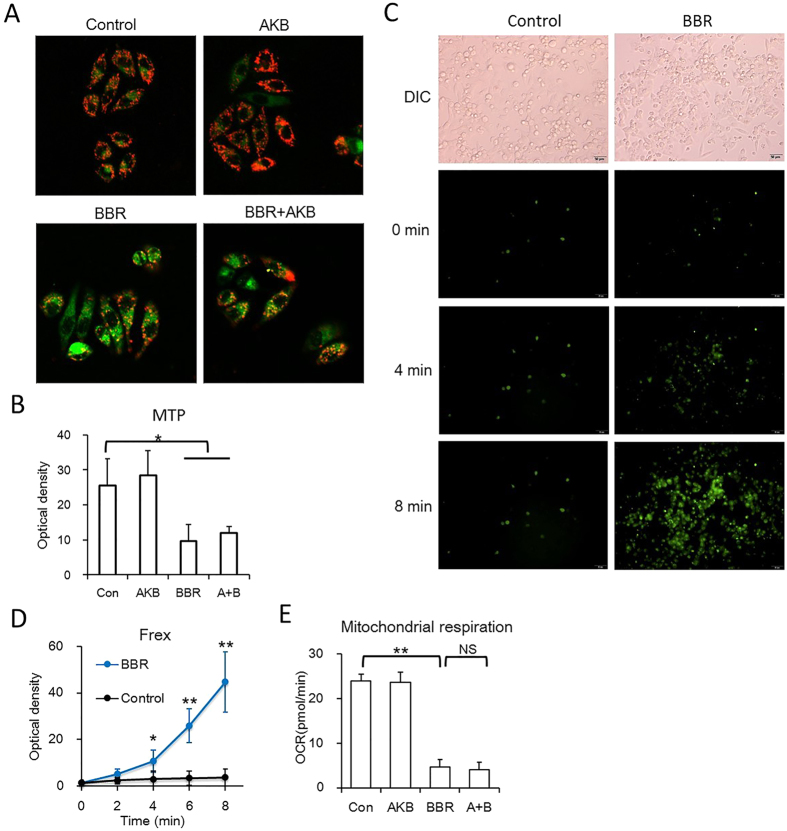
BBR reduces mitochondrial activity and AKB could not rescue that process. (**A**,**B**) BBR reduced mitochondrial membrane potential. HepG2 cells were stained by JC-10. (**A**) Representative confocal images (20×). (**B**) Statistical analysis of the optical density of the 590 nm of JC-10 aggregates. BBR 60 μmol/L, AKB 1 mmol/L. Mean ± SEM; *p < 0.05, **p < 0.01, n = 6. (**C,D**) BBR induced NADH concentration rise, indicated by Frex protein, a fluorescent biosensor of NADH. BBR 60 μmol/L. (**C**) Representative living cell images (20×). (**D**) Statistical analysis of the optical density of the NADH activated Frex protein. Mean ± SEM; *p < 0.05, **p < 0.01, n = 6. (**E**) Basal respiration of the HepG2 cells with the indicated treatment. The assay was conducted on a Seahorse XF Analyzer. BBR 60 μmol/L, AKB 1 mmol/L. Mean ± SEM; **p < 0.01, n = 3.

Mitochondria occur a series of dynamic changes in response to metabolic stresses, including depolarization, fragmentation, mitochondrial DNA copy number change and so on^19^. Previous studies have shown that BBR inhibited mitochondrial activity by reducing oxygen consumption^12^,^20^. Our results showed that, in consistent with reported researches, BBR inhibited mitochondrial activity by reducing mitochondrial transmembrane potential (MTP), and AKB had no significant impact on MTP (Fig. 3A and B). In addition, BBR also reduce oxygen consumption in HepG2 cells, and AKB had no significant impact on mitochondrial basal respiration (Fig. 3E). Here in our study, the NADH level was measured to determine the regulation of mitochondrial activity by BBR. Frex protein is a biosensor that binds to and is activated by NADH^21^. The results showed that BBR significantly increased the fluorescence of Frex protein within a few minutes (Fig. 3C and D), indicating that BBR quickly and significantly increased electron transport from NADH to its down-stream electron receptors.

**Figure 4:**
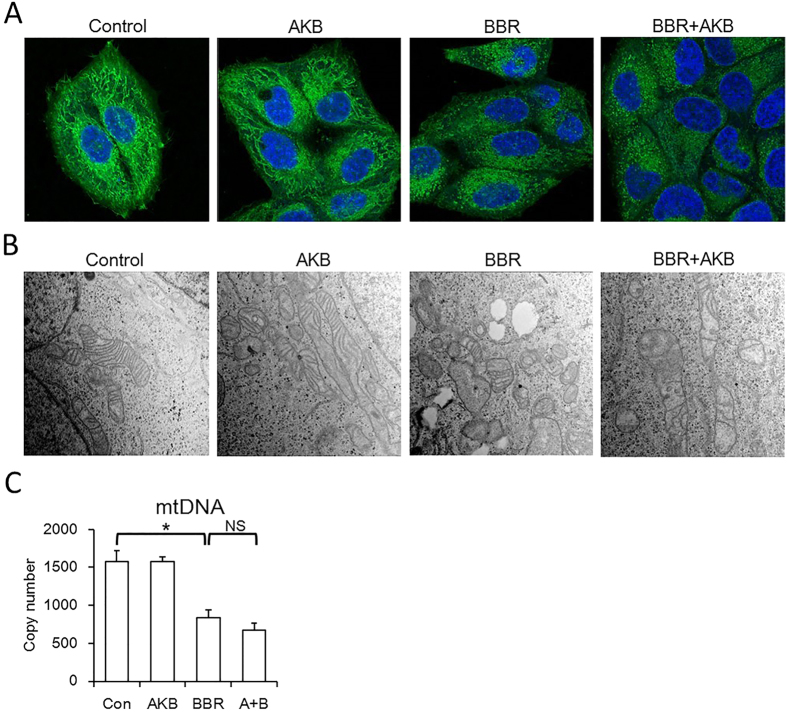
BBR damage mitochondrial structure and AKB could not rescue that process. (**A**) Representative confocal images (63×) showing BBR induced mitochondrial fission. Cells were stained by Mito-Tracker Green (MTG) and DAPI. BBR 60 μmol/L, AKB 1 mmol/L. (**B**) Representative transmission electron microscopy images (30000×) showing BBR induced mitochondrial vacuolation. BBR 60 μmol/L, AKB 1 mmol/L. (**C**) Mitochondrial DNA (mtDNA) copy number. Determined by real-time PCR. BBR 60 μmol/L, AKB 1 mmol/L. Mean ± SEM; *p < 0.05, n = 3.

After BBR treatment, mitochondria also underwent structure collapse. Mitochondrial network became disrupted and mitochondria underwent fission, and the mitochondrial cristae decreased and even disappeared, and AKB also did not recover that damage (Fig. 4A and B). Moreover, mitochondrial DNA (mtDNA) copy number, were reduced by BBR and not rescued by AKB (Fig. 4C). Taken together, our results suggest that BBR suppress mitochondrial activity and damage mitochondrial morphology, and AKB fails to prevent mitochondria from BBR-induced activity suppression and morphological changes.

### BBR diminishes TCA cycle metabolites and AKB does not recover that damage

The above results showed that a toxic dose of BBR inhibits glucose consumption and mitochondrial activity. The details of the inhibition were further investigated using a metabonomics approach. After the indicated treatment, the metabolites were harvested and detected by LC-MS/MS on a triple quadrupole mass spectrometer (Thermo, CA, USA). Data were analysed by TSQ Quantiva (Thermo, CA, USA). In addition, the NAD+ and NADH level were analysed by a LC-MS/MS analysis with standard.

**Figure 5:**
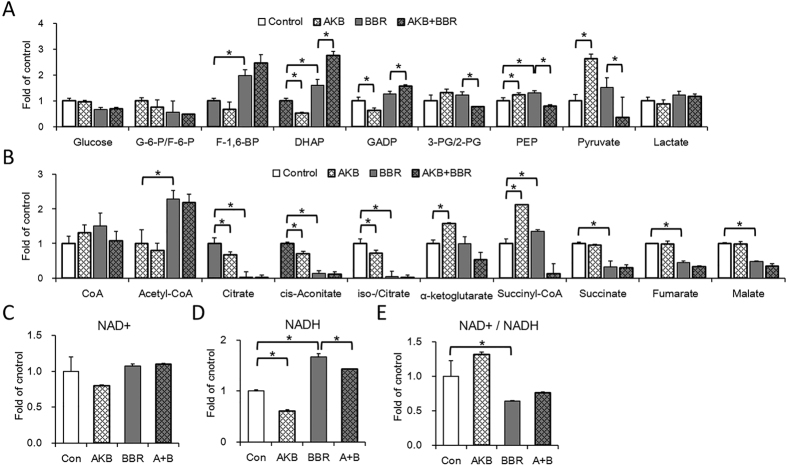
BBR increases glycolysis metabolites, decreases TCA cycle metabolites, reduces ratio of NAD+/NADH. Glycolysis and TCA cycle metabolites were quantified by a metabonomics analysis, NAD+ and NADH was quantified by LC-MS analysis. (**A**) Relative glycolysis metabolites concentration. (**B**) Relative TCA cycle metabolites concentration. HepG2 cells were cultured without or with AKB or/and BBR. (**C**) Relative NAD+ concentration. Con, control; BBR 60 μmol/L; AKB 1 mmol/L; A + B, BBR 60 μmol/L and AKB 1 mmol/L. Mean ± SEM; *p < 0.05, n = 3. (**D**) Relative NADH concentration. Con, control; BBR 60 μmol/L; AKB 1 mmol/L; A + B, BBR 60 μmol/L and AKB 1 mmol/L. Mean ± SEM; *p < 0.05, n = 3. (**E**) Relative NAD+/NADH ratio. Con, control; BBR 60 μmol/L; AKB 1 mmol/L; A + B, BBR 60 μmol/L and AKB 1 mmol/L. Mean ± SEM; *p < 0.05, n = 3.

All glycolysis metabolites were detected except for 1,3-bisphosphoglycerate (Fig. 5A). BBR up-regulated the concentration of fructose 1,6-bisphosphate (F-1, 6-BP), dihydroxyacetone phosphate (DHAP) and phosphoenolpyruvate (PEP). No metabolites were significantly down-regulated by BBR. G-6-P, F-1, 6-BP and pyruvate, which are products of the three rate-limiting steps of glycolysis, were not down-regulated by BBR. Although AKB down-regulated F-1, 6-BP, DHAP and GADP, whereas BBR up-regulated F-BP, DHAP and GADP, the BBR plus AKB group showed higher concentrations of F-1, 6-BP, DHAP and GADP compared to the BBR only group. Conversely, both AKB and BBR alone up-regulated PEP and pyruvate; however, the BBR plus AKB group showed lower concentrations of PEP and pyruvate.

For TCA cycle analysis, all metabolites in the TCA cycle were detected except for oxaloacetate (Fig. 5B). Acetyl-CoA and succinyl–CoA were up-regulated by both AKB and BBR. However, the other metabolites in the TCA cycle were significantly down-regulated by BBR and AKB did not rescue that damage. Therefore, consistent with the changes in mitochondrial morphology, BBR destroyed the TCA cycle, and AKB did not rescue that damage. Additionally, the increased level of acetyl-coA would be a result of blocked TCA cycle.

For the NAD+ and NADH level, the results showed that although no significant NAD+ level change was detected in the study (Fig. 5C), however, BBR increased whilst AKB decreased NADH level, so did the NAD+/NADH ratio (Fig. 5D and E). The results suggested that the NAD+/NADH balance was interrupted by BBR administration, which is in consistent with the Frex biosensor experiment (Fig. 3C) and other experiments in this study.

Compared with the glycolysis metabolites, the TCA cycle metabolites change more significantly. Thus BBR more likely to target on mitochondria. And AKB recovers cell proliferation not via protect TCA cycle metabolism from BBR inhibition.

### Different from rotenone, a low dose of BBR reduces mtDNA copy number

**Figure 6:**
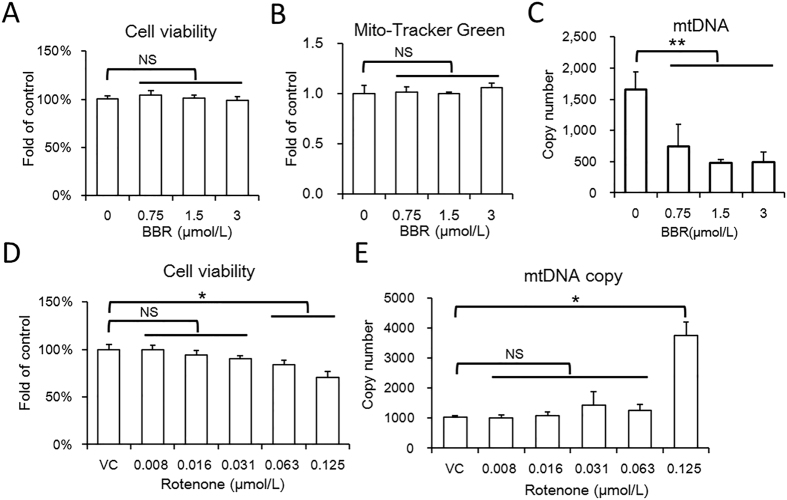
Safety dose of BBR reduce mtDNA copy number. (**A**) CCK-8 assay of HepG2 cell treated with BBR. Mean ± SEM; NS no significant difference, n = 6. (**B**) Mitochondrial biomass of HepG2 cells treated with BBR at the indicated dose. Mitochondrial biomass was determined by MTG assay on a high content system. Mean ± SEM; n = 6. (**C**) The mtDNA copy number of HepG2 cells with BBR treatment at the indicated doses. Determined by relative quantitative PCR, the genome DNA served as reference. Mean ± SEM; **p < 0.01, n = 3. (**D**) CCK-8 assay of HepG2 cell treated with BBR. Mean ± SEM; NS no significant difference, *p < 0.05, n = 6. (**E**) The mtDNA copy number of HepG2 cells with rotenone treatment at the indicated doses. Mean ± SEM; *p < 0.05, n = 3.

Both BBR and rotenone have been found to reduce mitochondrial activity via inhibiting respiration complex I, and rotenone has served as a positive control in some previous studies on BBR^20^. However, our study found a difference in the mtDNA copy number regulation between the two drugs. BBR showed no significant reduction on mitochondrial activity or cell viability in HepG2 cells when using less than 3 μmol/L (Fig. 6A, S1). Within this safety dose, BBR also showed no significant regulation on mitochondrial biomass, as indicated by Mito-Tracker Green straining (Fig. 6B). However, BBR significantly reduced the mtDNA copy number (Fig. 6C), indicating that the mtDNA copy number is sensitive to BBR. Different from BBR, rotenone showed no significant effect to mtDNA copy number at safety doses and up-regulated mtDNA copy number at toxic doses (Fig. 6D and E). Therefore, BBR and rotenone differ in their actions on mitochondrial activity. The mechanism by which BBR reduces mtDNA copy number requires further study.

### BBR reduces tumour growth and mitochondrial activity *in vivo*

As BBR regulates glucose metabolism, cell proliferation and mitochondrial activity in cultured HepG2 cells, the use of BBR on tumours *in vivo* is attractive. Thus, a mouse xenograft model was introduced to test the anticancer effect of BBR. The tumour was introduced by injecting HepG2 cells subcutaneously in the backs of mice that were orally administered BBR every day at the indicated dose for one month.

**Figure 7:**
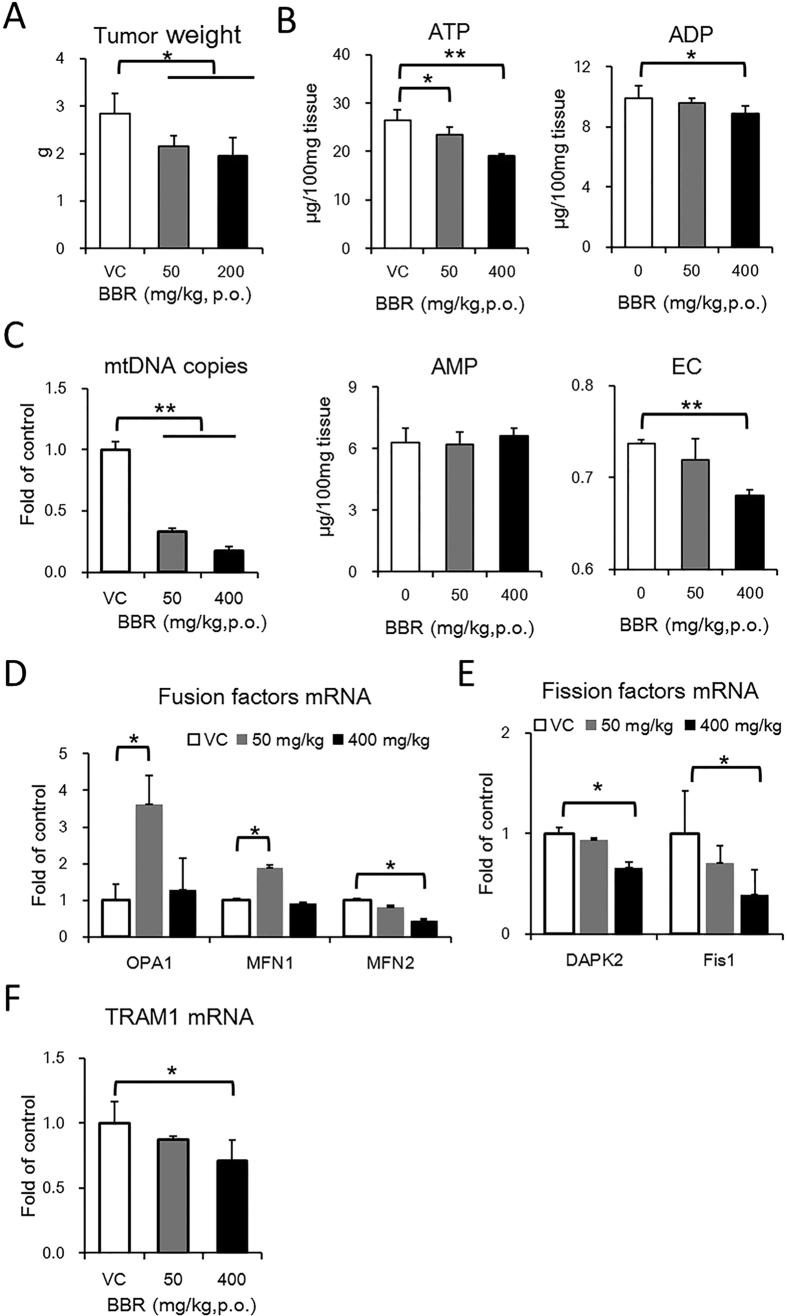
BBR suppresses tumor growth and inhibits mitochondrial activity. HepG2 xenograft model was established by subcutaneously implanting HepG2 cells into CD1-Nude mice. And the mice were given vehicle or BBR at the indicated doses for 4 weeks. N = 6. (**A**) Final tumor weight at the end of experiment. Mean ± SEM; *p < 0.05, n = 6. (**B**) ATP, ADP and AMP concentration and energy charge (EC) of tumor. Mean ± SEM; *p < 0.05, **p < 0.01, n = 6. (**C**) The mRNA expression of enzymes in glycolysis. Mean ± SEM; *p < 0.05, ***p < 0.001, n = 3. (**D**) mRNA expression of TCA cycle enzymes. Mean ± SEM; *p < 0.05, n = 3. (**E**) mtDNA copy number of tumor cell. Mean ± SEM; **p < 0.01, n = 3. (**F**) mRNA expression of factors involved in mitochondrial fusion. Mean ± SEM; *p < 0.05, n = 3. (**G**) mRNA expression of factors involved in mitochondrial. Mean ± SEM; *p < 0.05, n = 3. (**H**) mRNA expression of factors involved in mtDNA replication. Mean ± SEM; *p < 0.05, n = 3.

The results showed that both 50 mg/kg and 400 mg/kg of BBR suppressed tumour growth *in vivo* (Fig. 7A). However, BBR showed only a 30% reduction of tumour weight, and no significant dose dependent effect was observed. No mice died during BBR administration, but BBR retarded mice body weight gain (Figure S2). In tumour cells, the mRNA expression of glycolytic enzymes was generally up-regulated by BBR, and the mRNA expression of TCA cycle enzymes was generally down-regulated by BBR (Fig. 7C and D). In addition, our results indicated that BBR reduces the level of ATP and energy charge (Fig. 7B). These results are consistent with the decreased glucose consumption and reduced TCA cycle activity by BBR *in vitro*.

Mitochondria undergoes fusion and mtDNA increases in response to the a lowered energy status, whilst the mitochondrial fission and mitochondrial DNA (mtDNA) number decrease occurs in a high-energy status^22^,^23^,^24^. According to our results, the mtDNA copy number of tumour cells was reduced by BBR in a dose-dependent manner (Fig. 7E). Mitochondrial fusion factors optic atrophy 1 (OPA1) and mitofusin-1 (Mfn1) were up-regulated by 50 mg/kg BBR; however, 400 mg/kg BBR showed no significant difference, whilst mitofusin-2 (Mfn2) was reduced by BBR in a dose-dependent manner (Fig. 7F). Additionally, the fission factors death-associated protein kinase 2 (DAPK2) and fission-1 (Fis1) were down regulated by BBR (Fig. 7G). The mitochondrial transcription factor TFAM, which is responsible for mtDNA replication^25^, was also reduced by BBR in a dose-dependent manner (Fig. 7H), In summary, BBR reduces mitochondrial activity in general, and BBR regulated mitochondrial dynamics factors in a complicated mode.

## Discussion

Identifying the decisive target of BBR is a great challenge in the pharmacological analysis of BBR. Previous studies have identified a lot of targets of BBR, and many studies have revealed that mitochondrial is one target of BBR^15^,^26^, but no study has designed experiment to figure out convincingly which target played a decisive role. Here in our study, we make a step by designing an experiment.

**Figure 8:**
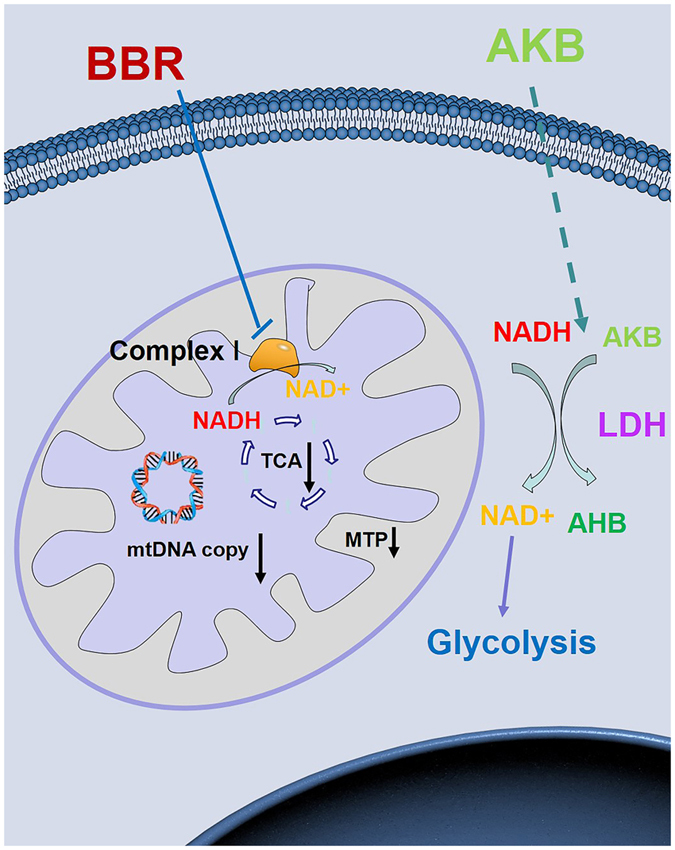
BBR inhibits mitochondrial activity and exogenous electron receptor help to regenerate NAD+. BBR administration inhibits mitochondrial respiration, reduces mitochondrial transmembrane potential (MTP). In addition, the NAD+ regeneration is blocked and TCA cycle is inhibited by BBR either. Exogenous electron receptor like pyruvate and AKB can serves as the substrate of LDH and help to regenerate NAD+. Thus, exogenous electron receptor can make up for the reduced mitochondrial activity in BBR administration.

In this study, a rescue study was utilized to show the important role of mitochondria in BBR activity. The principle of this rescue study is that, if a target play a primary role in a signal pathway or metabolic process, then inhibits of this target would block this pathway and recover the function of this target by some method would abolish the inhibition. Our results show that BBR inhibited cell proliferation and mitochondrial activity, and AKB partially restores the impaired cell proliferation but does not prevent mitochondrial damage (Fig. 8). Compared to the control group, AKB recovered no less than half of the proliferation rate in different cells. AKB helps regenerate NAD+, which partially substitutes the function of mitochondria. As AKB did not directly interact with any targets of BBR and only served as a substrate of LDH and supported the regeneration of NAD+, which is part of the function of mitochondria. The inhibition of the cell proliferation is the combined effects of all the targets of BBR, among all these targets, which one is the main targets of BBR is debatable and no direct experiment could solve this problem. Here in our study, the rescue study was able to distinguish mitochondria from other BBR targets, and the recovery of the cell proliferation ratio indicates the contribution of mitochondria to the integral activity of BBR. As AKB restored cell proliferation by no less than a half, thus it can be concluded that mitochondria play a decisive role in the inhibition of cell proliferation by BBR.

Cell proliferation was chosen as the indicator of the effect of BBR because cell proliferation requires the duplication of all cell components and most metabolic pathways in the cells involved. Different doses of BBR may exert opposite regulatory effects on some metabolic and signalling pathways; however, the direct action of BBR on its targets would be consistent amongst the different doses. Therefore, a dose of BBR that completely inhibits cell proliferation was utilized to magnify the direct action of BBR on all of its potential targets.

Our study also provides insights into how BBR regulates cell metabolism. Previous studies have shown that BBR blocks respiration complex I by measuring oxygen consumption^11^,^12^. In our study, we directly demonstrate that BBR raised NADH concentration within a few minutes. Moreover, our results show that BBR reduces mtDNA copy number at a non-toxic dose, whilst the NADH dehydrogenase inhibitor rotenone showed no significant regulation. This difference suggests that the mechanism by which BBR inhibits mitochondrial activity may not be identical to that of rotenone, and further study is needed to determine how BBR down-regulates mtDNA copy number.

BBR may serve as an adjuvant therapy by reducing mitochondrial activity. Cell proliferation and metabolism require mitochondria to provide metabolites and to support NAD+ regeneration^17^,^27^, Mitochondria are overburdened in tumour cells as tumour cells proliferate quickly; therefore, the tumour cell is more sensitive to mitochondria-inhibiting drugs than normal cells^28^,^29^, and BBR is one of these mitochondria inhibiting drugs. BBR also up-regulated glycolysis enzymes expression which is similar to Warburg effect. As cancer cell is characterized by Warburg effect, the role of BBR needs further research^18^.

The rescue experiment utilized in our study could be introduced for the pharmacological analysis of many other natural compounds to examine the role of mitochondria in their integral activity. In addition to BBR, other alkaloids are found to exhibit anti-cancer effects, and the pharmacological study find these drugs accelerate in mitochondria depending on mitochondria transmembrane potential and have various targets in cell^30^. The recovery ratio of cell proliferation could be determined in such drugs to show the contribution of mitochondria to their anti-cancer effects.

## Methods

### Chemicals and kits

Berberine hydrochloride (>98%) was purchased from Beijing Shuanghe Pharmacy (Beijing, China). α-Ketobutyrate (AKB), Oligomycin, FCCP (Carbonyl cyanide-p-trifluoromethoxyphenylhydra-zone), Rotenone, Antimycin, ATP, ADP and AMP were purchased from Sigma-Aldrich (St. Louis, MO, USA). α-Hydroxybutyrate was purchased from Alfa Aesar (Heysham, UK). CCK-8 and Mito-Tracker Green were purchased from Beyotime Biotechnology (Shanghai, China). All assays were conducted according to the manufacturer’s instructions.

### Animals and ethical statement

CD1-nude mice (male, 4 weeks old, 16–18 g) used in the study were purchased from Vital River (Beijing, China) and maintained under specific pathogen free (SPF) conditions in the Laboratory Animal Research Center of Tsinghua University. The laboratory animal facility has been accredited by the AAALAC (Association for Assessment and Accreditation of Laboratory Animal Care International). All experimental procedures were approved by the IACUC (Institutional Animal Care and Use Committee) of Tsinghua University and were performed in accordance with the People’s Republic of China Legislation Regarding the Use and Care of Laboratory Animals (Approval ID: 15-DLJ2).

### Cell lines

HepG2, HeLa and Hepa1-6 cells were cultured in Dulbecco’s Modified Eagle’s Medium (DMEM) (Gibco, CA, USA) supplemented with 10% foetal bovine serum (FBS) (HyClone, UT, USA), 100 U/ml penicillin and 100 mg/ml streptomycin. C17.2 cells were cultured in DMEM supplemented with 10% FBS, 5% horse serum, 100 U/ml penicillin and 100 mg/ml streptomycin. The DMEM did not include pyruvate. All cells were incubated at 37 °C with 5% CO_2_.

### Cell proliferation rate

Cells were seeded in 6-well dishes (Corning, NY, USA) and incubated overnight. One well from each group was harvested and counted with a haemocytometer to determine the initial cell number at the time of treatment. Cells were washed with phosphate-buffered saline (PBS) and given the drug treatment as indicated. The medium was refreshed every 24 h. The cell proliferation rate was determined using the following formula: proliferation rate (doublings per day) = log2 (today’s cell number/yesterday’s cell number). The average cell proliferation rate represents the average value over the last three days.

### Glucose consumption

Glucose consumption is defined as the difference between the initial glucose concentration and the terminal glucose concentration in the medium. Glucose concentration was determined by a Glucose Assay Kit (BIOSINO, Beijing, China).

### LC-MS/MS analysis for AKB and AHB

Cells were seeded in 6-well plate and cultured overnight. Then cell medium was refreshed and cells were cultured with 1 mM AKB and 60 μmol/L BBR for the indicated times. Then the medium was extracted with 80% methanol at the indicated time after treatment. After centrifugation, the supernatants were dried and dissolved in methanol. The identification and quantification of analytes were carried out on an Agilent 1290 (Agilent, CA, USA) liquid chromatography equipped with a Agilent 6460 QQQ (Agilent, CA, USA) tandem mass spectrometer in negative ion mode. A Synergi 4 μ hydro-RP, 2.0 mm × 150 mm (Phenomenex, CA, USA) was used for LC separation. Solvent A: methanol; Solvent B: 5 mM tributylamine, 15 mM acetic acid and 3% methanol. The gradient programme used was as follows: 0–3 min 0% A, 3–8 min 0% A to 10% A, 8–13 min 10% A to 30% A, 13–20 min 30% A to 60% A. 20.1–26 min 0% A. The flow rate was set at 0.3 ml/min, and the injection volume was 10 μL. The total run time for each sample was 26 min. The concentration of AKB and AHB was determined by a standard curve. AKB and AHB standard was purchased from Sigma (MO, USA), and N,N-Dimethylformamide (Sigma, MO, USA) served as the internal standard which was added in the medium before 80% methanol extraction.

### LC-MS/MS analysis for NAD+ and NADH

Cells were seeded in 10 cm dishes and cultured overnight. Then cell medium was refreshed and cells were cultured with the indicated treatment for 2 h. Then the medium was removed and the cells were washed by pre-cold PBS twice and extracted with pre-cold methanol. The extract was centrifuged and the supernatants were dried and dissolved in deionized water. Liquid chromatography was performed on a rapid resolution liquid chromatography system (Nexera UHPLC LC-30A, Shimadzu, Japan) with a VanGuard 1.8 μm HSS T3, 2.1 mm × 100 mm column (Waters, MA, USA). Solvent A: 10 mmol/L ammonium acetate, 0.01% NH_3_• H_2_O in Acetonitrile; Solvent B: 10 mmol/L ammonium acetate solution and 0.01% NH3 in water. The gradient programme used was as follows: 0–2 min 0% A, 2–4 min 0% A to 10% A, 4–5 min 10% A-100% A, 5–6 min 100% A, 6.1–10 min 0% A. The flow rate was set at 0.2 ml/min, and the injection volume was 10 μL. The total run time was 10 min for each sample. The detection was performed on an AB SCIEX Triple Quad™ 4500 (Applied Biosystems,Foster City, CA, USA) with an electrospray ionization source (Turbo Ionspray). The mass spectrometry detection was operated in negative electrospray ionization mode. NAD+ and NADH standard was purchased from Sigma (MO, USA).

### Mitochondrial transmembrane potential assay

Cells were seeded in confocal culture dishes and cultured overnight. After that, cells were stained with JC-10 following the manufacturer’s instruction (Solarbio, Beijing, China). Then, the samples were tested on a Confocal Laser Scanning Microscope (Zeiss LSM710, Oberkochen, Germany). The statistical analysis of the optical density is performed by Fuji software^31^.

### NADH determination by the fluorescence biosensor Frex

Cells were seeded in 3.5 cm dishes and cultured overnight and cells were transfected with Frex plasmids. 24 h after transfection, cells were loaded on a Live cell Imaging System (Olympus IX81, Tokyo, Japan). Then cells were administered BBR at the indicated dose or a vehicle control (ddH_2_O). Frex plasmids were provided by Doctor Ye-Yang from ECUST (East China University of Science and Technology). The statistical analysis of the optical density is performed by Fuji software^31^.

### Mito-Tracker Green Staining

HepG2 cells were seeded in 3.5 cm dishes and cultured overnight. Then, cells were treated with BBR or AKB for 24 h as indicated dose. After that, the medium was removed and cells were fixed with 4% formaldehyde for 10 min, then formaldehyde was removed and cells were washed by PBS twice. Cells were double stained with 200 nmol/L Mito-Tracker Green and 1 μg/ml 4′,6-diamidino-2-phenylindole (DAPI) for 10 min. Then cells were washed by PBS. After staining, cells were imaged by a Confocal Laser Scanning Microscope (Zeiss LSM780, Oberkochen, Germany). The image was blind deconvoluted three times with an autoquant software (Media cybemetics).

### Mitochondrial transmembrane potential assay

HepG2 cells were seeded in 3.5 cm dishes and cultured overnight. And cells were stained with JC-10 (KeyGEN BioTECH, Beijing, China) following the protocol. After staining, cells were administrated with BBR or AKB as indicated for 2 h. Then cells were put on a Confocal Laser Scanning Microscope (Zeiss LSM710, Oberkochen, Germany) with Living cell system and imaged.

### Transmission electron microscopy

After the indicated drug treatment, cells were harvested with trypsin and fixed with 2.5% glutaraldehyde in 0.2 mol/L phosphate buffer (pH 7.4) for 2 h. Cells were then washed with 0.1 mol/L phosphate buffer three times at 10 min each. After centrifugation, the precipitate was resuspended in 1% OsO4 buffer and incubated for 1.5 h. Cells were then washed once more with 0.1 mol/L phosphate buffer. Samples were dehydrated in ethanol, embedded in Spurr’s resin and solidified in an oven. Ultrathin sections were viewed on an EM UC6 ultramicrotome (Leica, Germany) at a thickness of 70 nm. The sections were double stained with 3% uranyl acetate and lead citrate, and examined on a H-7650B electron microscope (HITACHI, Tokyo, Japan).

### Mitochondrial respiration assay

HepG2 cells were seeded in XF 96-well microplate (Seahorse biosciences, MA, USA) at 10^4^ cells/well in 80 μl of growth medium and cultured overnight. Next day, cells were washed and refreshed with assay medium (Base medium, 4.5 g/L glucose and 584 mg/ml glutamine) with the indicated drugs. Then the cells were incubated at 37 °C without CO_2_ for 1 h. After preequilibration, the microplate was put in a Seahorse XFe96 Analyzer (Seahorse biosciences, MA, USA), and a mito-stress assay was conducted. Reagents were used as follows: 2 μmol/L Oligomycin, 2 μmol/L FCCP, 1 μmol/L Rotenone and 1 μmol/L Antimycin.

### CCK-8 assay

HepG2 cells were seeded in 96-well plates at a concentration of 10,000 cells per well and cultured overnight. The medium was refreshed and cells were incubated with BBR at the indicated doses for 24 h. The medium was then replaced with fresh medium and 10 μL of CCK-8 solution was added to each well. After incubation for 20 min, the absorption was measured at 450 nm on a microplate reader (Bio-Rad, CA, USA) following the manufacturer’s protocol.

### High content screening

HepG2 cells were seeded in 96-well plates (PerkinElmer CellCarrier, CA, USA) and cultured overnight. Then, cells were treated with BBR gradient for 24 h. After that, the medium was removed and cells were fixed with 4% formaldehyde for 10 min, then formaldehyde was removed and cells were washed by PBS twice. Cells were double stained with 200 nmol/L Mito-Tracker Green and 1 μg/ml 4′,6-diamidino-2-phenylindole (DAPI) for 10 min. Then cells were washed by PBS. After staining, cells were analyzed by a High Content Screening System (PerkinElmer Opera Phenix, CA, USA). The mitochondrial biomass is defined as cytoplasmic MTG light intensity per cell.

### Metabolomic experiment

HepG2 cells were seeded onto 10 cm wells and cultured overnight. Cell medium was refreshed and cells were given the indicated drugs for 8 h. The medium was then removed and cells were washed with pre-chilled PBS buffer. Thereafter, 2 ml of 80% methanol (−20 °C pre-chilled) was added and incubated at −20 °C for 1 h. The solutions were harvested and centrifuged and the supernatants dried with rotary evaporators and dissolved. The targeted metabolomic experiment was analysed by TSQ Quantiva (Thermo, CA, USA). C18-based reverse phase chromatography was utilized with 10 mM tributylamine, 15 mmol/L acetate in water and 100% methanol as mobile phase A and B, respectively. This analysis focused on the TCA cycle, glycolysis, the pentose phosphate pathway, and amino acid and purine metabolism. In this experiment, we used a 25-min gradient from 5% to 90% mobile B. Positive-negative ion switching mode was used for data acquisition. The cycle time was set as 1 s and a total of 138 ion pairs were included. The resolution for Q1 and Q3 were both 0.7 full width at half maximum (FWHM). The source voltage was 3500 V for positive ion mode and 2500 V for negative ion mode. Sweep gas was turned on at a flow rate of 1 (arb).

### HepG2 xenograft model

Briefly, CD1-nude mice were injected subcutaneously with HepG2 cells at a concentration of 10^6^ cells per mouse. After one week of feeding, the mice successfully seeded with a tumour were selected for BBR administration. Mice were orally administered an equal amount of distilled water, 50 mg/kg BBR or 400 mg/kg BBR for one month. Mice were killed by cervical dislocation and the tumour tissues were separated for the following assay. All mice were allowed food and drink ad libitum, and no mouse died during the study.

### HPLC for ATP, ADP and AMP test

Fresh mice liver tissue was added pre-cooling 0.5 mol/L HClO_4_ at the ratio of 100 mg tissue per 1 ml solution. Then the tissue was homogenized completely and centrifuged (4 °C, 12000 g, 10 min). The supernatant was added 1 mol/L KOH solution at the termination of pH = 6.5. The mixture was centrifuged (4 °C, 12000 g, 10 min) again and the supernatant was tested on Agilent 1260 high resolution liquid chromatography system (CA, USA) with a UV-absorbance detector (254 nm). The column was Thermo Hypersil Gold 4 μm 250 mm × 4.6 mm and the mobile phase was 0.1 mol/L potassium phosphate buffer (pH = 6.5). The flow rate was 1.0 ml/min and the injection volume was 10 μL. The total run time was 15 min for each sample. ATP, ADP and AMP standard was purchased from Sigma (MO, USA).

### RNA extraction and Real-time PCR

The fluorescence analysis was performed as described previously^32^. Briefly, total RNA was extracted from tumours using an RNA pre-pure Cell/Bacteria Kit (Tiangen Biotech, Beijing, China) and reverse-transcribed into cDNA using a FastQuant RT Kit (Tiangen Biotech, China) according to the manufacturer’s instructions. Real-time PCR for the indicated genes was detected on an LC480 (Roche, Basel, Germany) with a SYBR Green Master Mix Kit (Tiangen Biotech, Beijing, China) following the manufacturer’s instructions. The primers used in the experiments are described in Table S1. β-actin mRNA served as an internal reference, and the results were normalized and are shown as the ratio to the control group.

### mtDNA copy number determination

Both of the genome DNA and mtDNA were extracted simultaneously with a genome DNA extraction kit (Tiangen Biotech, Beijing, China) following the manufacture’s protocol (The genome DNA extraction kit is able to extract both genome DNA and mtDNA according to the information provided by the manufacture). Then the mtDNA copy number per cell is determined by Real-time PCR. The mixture of genome DNA and mtDNA served as the template, and the primer for genome DNA and mtDNA were listed in Table S1. mtDNA sequence served as the target while genome DNA sequence served as the reference.

### Statistical analysis

Results are expressed as the mean ± standard error of the mean (SEM). Student’s *t*-tests for statistical analysis using Excel Software for Office 2013 (Microsoft, USA) was performed for all data except for tumour weight data. And tumour weight data was statistically analyzed using one-way analysis of variance (ANOVA) by SPSS software (IBM, USA). The data passed the normality test and the homogeneity test of variance. NS was considered to be not statistically significant, P < 0.05 was considered to be statistically significant, P < 0.01 was considered to be very statistically significant, P < 0.001 was considered to be highly statistically significant.

## Additional Information

**How to cite this article:** Yan, X.-J. *et al*. Mitochondria play an important role in the cell proliferation suppressing activity of berberine. *Sci. Rep.* **7**, 41712; doi: 10.1038/srep41712 (2017).

**Publisher's note:** Springer Nature remains neutral with regard to jurisdictional claims in published maps and institutional affiliations.

## Supplementary Material

Supplementary Information
