# Supplementary material for: Mitochondria play an important role in the cell proliferation suppressing activity of berberine
Source: Sci Rep. 2017 Feb 9;7:41712. doi: 10.1038/srep41712 (PMC5299431; doi:10.1038/srep41712)
Supplement: Supplementary Information [file srep41712-s1.pdf]

## Supplementary information

# Mitochondria play an important role in the cell proliferation suppressing activity of berberine

Xiao-Jin Yan†, Xuan Yu†, Xin-Pei Wang†, Jing-Fei Jiang, Zhi-Yi Yuan, Xi Lu, Fan Lei, Dong-Ming Xing*

MOE Key Laboratory of Protein Sciences, Laboratory of Molecular Pharmacology and Pharmaceutical Sciences, School of Life Sciences, Tsinghua University, Beijing 100084, China

† Equal in contribution

To whom correspondence should be addressed:

Dong-Ming Xing

MOE Key Laboratory of Protein Sciences, Laboratory of Molecular Pharmacology and Pharmaceutical Sciences, School of Life Sciences,

Tsinghua University,

Beijing 100084, China

Tel:+86-10-62796270

E-mail: pharm@mail.tsinghua.edu.cn

# Supplementary Information

**Figure S1. Dose of BBR or rotenone for HepG2 cell viability.** Cell number was determined with a hemocytometer. **(A).** HepG2 cell number after BBR treatment at the indicated dose for 24 h. Mean ±SEM; NS no significant difference, n = 3. **(B).** HepG2 cell number after rotenone treatment at the indicated dose for 24 h. Mean ±SEM; NS no significant difference, n = 3.

**Figure S2. Mice body weight gain in the HepG2 xenograft model.** (A). Mice body weight gain in the HepG2 xenograft model. Mean ±SEM; *$p < 0.05$, n = 6.

**Table S1. Primer sequences used in the experiment**

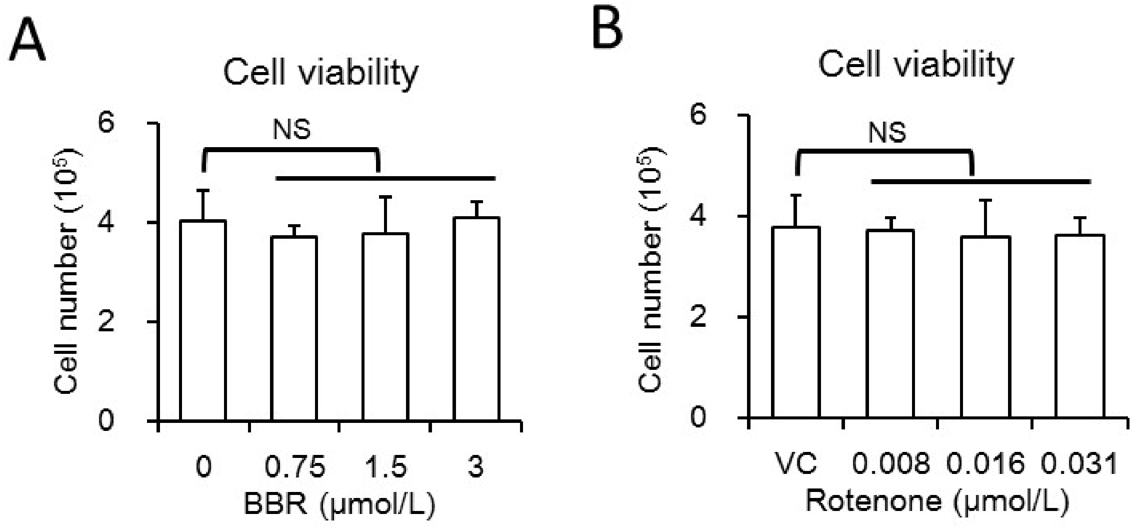


**Figure S1. Dose of BBR or rotenone for HepG2 cell viability.** Cell number was determined with a hemocytometer. **(A).** HepG2 cell number after BBR treatment at the indicated dose for 24 h. Mean ±SEM; NS no significant difference, n = 3. **(B).** HepG2 cell number after rotenone treatment at the indicated dose for 24 h. Mean ±SEM; NS no significant difference, n = 3.

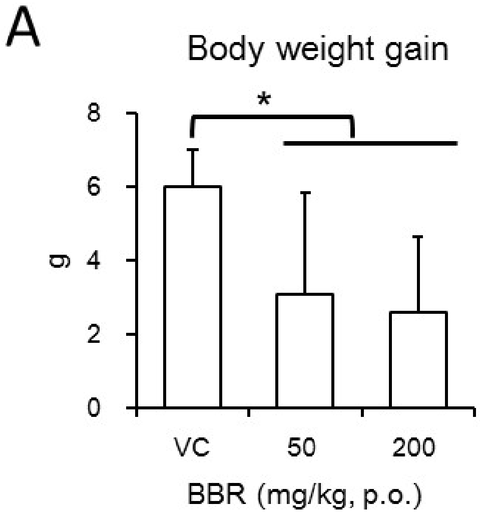


**Figure S2. Mice body weight gain in the HepG2 xenograft model. (A).** Mice body weight gain in the HepG2 xenograft model. Mean ±SEM; $*p < 0.05$, $n = 6$.

**Table S1. Primer sequences used in the experiment**

| Targeted sequence | Primer sequence | Product size(bp) |
|---|---|---|
| Human genome DNA | Sense 5’- TGCTGAGTCACCTTCGAACC-3’ | 562 |
| | Anti-sense 5’- AGCATAACCTGACACCAGCC-3’ | |
| Human mtDNA | Sense 5’- ACAATTCTCCGATCCGTCCC-3’ | 136 |
| | Anti-sense 5’- GTGATTGGCTTAGTGGGCGA-3’ | |
| Mouse genome DNA | Sense 5’- CCTATCAGTTCGGAGCCCAC-3’ | 586 |
| | Anti-sense 5’- TGGAACTCACCCGTTCACAC-3’ | |
| Mouse mtDNA | Sense 5’- AGCTCAATCTGCTTACGCCA-3’ | 150 |
| | Anti-sense 5’- TGTGAGGCCATGTGCGATTA-3’ | |
| Human β-actin | Sense 5’- AGGCTGGTCCTCCTATCGTT-3’ | 201 |
| | Anti-sense 5’- AGGCTGGTCCTCCTATCGTT-3’ | |
| Human OPA1 | Sense 5’- GTGGTTGGAGATCAGAGTGCTG-3’ | 130 |
| | Anti-sense 5’- GAGGACCTTCACTCAGAGTCAC-3’ | |
| Human Mfn1 | Sense 5’- GGTGAATGAGCGGCTTTCCAAG-3’ | 135 |
| | Anti-sense 5’- TCCTCCACCAAGAAATGCAGGC-3’ | |
| Human Mfn2 | Sense 5’- ATTGCAGAGGCGGTTCGACTCA-3’ | 104 |
| | Anti-sense 5’- TTCAGTCGGTCTTGCCGCTCTT-3’ | |
| Human DAPK2 | Sense 5’- GCACCACAATGTCATCACGCTG-3’ | 125 |
| | Anti-sense 5’- CCTCACTCAGTGACTCCTTCTG-3’ | |
| Human Fis1 | Sense 5’- CAAGGAACTGGAGCGGCTCATT-3’ | 124 |
| | Anti-sense 5’- GGACACAGCAAGTCCGATGAGT-3’ | |
| Human TFAM | Sense 5’- GAGCGTGCTAAAAGCACTGG -3’ | 238 |
| | Anti-sense 5’- CCACAGGGCTGCAATTTTCC -3’ | |
| Human HK2 | Sense 5’- GAGTTTGACCTGGATGTGGTTGC-3’ | 130 |
| | Anti-sense 5’- CCTCCATGTAGCAGGCATTGCT-3’ | |
| Human GCK | Sense 5’- GGCCACCAAGAAGGAAAAGGT-3’ | 254 |
| | Anti-sense 5’- CCTCTCCCACTTTGACCAGCA-3’ | |
| Human PGI | Sense 5’- GTGTACCTTCTAGTCCCGCC-3’ | 159 |

| | | |
|---|---|---|
| | Anti-sense 5’- TGGTGTTGAGGGTCAAGCTG-3’ | |
| Human PFK1 | Sense 5’- AAGAAGTAGGCTGGCACGACGT-3’ | 113 |
| | Anti-sense 5’- GCGGATGTTCTCCACAATGGAC-3’ | |
| Human ALDO1 | Sense 5’- CCACGAGACCCTCTACCAGA-3’ | 253 |
| | Anti-sense 5’- GATAGCGAGGCTGGATGGAC-3’ | |
| Human TPI | Sense 5’- AACTGGAAGATGAACGGGCG-3’ | 143 |
| | Anti-sense 5’- TTGGGATCTAGCTTCTGCCG-3’ | |
| Human GADPH | Sense 5’- CTCTGCTCCTCCTGTTCGAC-3’ | 121 |
| | Anti-sense 5’- GCGCCCAATACGACCAAATC-3’ | |
| Human PGK1 | Sense 5’- CCACTGTGGCTTCTGGCATA-3’ | 166 |
| | Anti-sense 5’- ATGAGAGCTTTGGTTCCCCG-3’ | |
| Human PGAM1 | Sense 5’- GCTCTGCCCTTCTGGAATGAAG-3’ | 155 |
| | Anti-sense 5’- ATACCAGTCGGCAGGTTCAGCT-3’ | |
| Human Eno1 | Sense 5’- AGTCAACCAGATTGGCTCCGTG-3’ | 130 |
| | Anti-sense 5’- CACAACCAGGTCAGCGATGAAG-3’ | |
| Human PKLR | Sense 5’- CGGAAGGACACGGCATCAAGAT-3’ | 150 |
| | Anti-sense 5’- GAGCCAGGAAAACCTTCTCTGC-3’ | |
| Human PKM2 | Sense 5’- AATGCAGTCCTGGATGGAGC-3’ | 228 |
| | Anti-sense 5’- ACTGCAGCACTTGAAGGAGG-3’ | |
| Human LDHα | Sense 5’- AGCTGTTCCACTTAAGGCCC-3’ | 119 |
| | Anti-sense 5’- AGGAATCGGGAATGCACGTC-3’ | |
| Human PDHA | Sense 5’- GGATGGTGAACAGCAATCTTGCC-3’ | 148 |
| | Anti-sense 5’- TCGCTGGAGTAGATGTGGTAGC-3’ | |
| Human Cs | Sense 5’- CACAGGGTATCAGCCGAACCAA-3’ | 128 |
| | Anti-sense 5’- CCAATACCGCTGCCTTCTCTGT-3’ | |
| Human Aco2 | Sense 5’- CAATCGTCACCTCCTACAACAGG-3’ | 133 |
| | Anti-sense 5’- GTCTCTGGGTTGAACTTGAGGG-3’ | |
| Human IDH3A | Sense 5’- TCGGTGTGACACCAAGTGGCAA-3’ | 100 |
| | Anti-sense 5’- TTCGCCATGTCCTTGCCTGCAA-3’ | |
| Human OGDH | Sense 5’- GAGGCTGTCATGTACGTGTGCA-3’ | 140 |

| | | |
|---|---|---|
| | Anti-sense 5'- TACATGAGCGGCTGCGTGAACA-3' | |
| Human SUCLG1 | Sense 5'- CAAACTCGTTGGAGGAACCACTC-3' | 138 |
| | Anti-sense 5'- GCAGCAAAAGGCGGAGGAACAT-3' | |
| Human SDHA | Sense 5'- GAGATGTGGTGTCTCGGTCCAT-3' | 142 |
| | Anti-sense 5'- GCTGTCTCTGAAATGCCAGGCA-3' | |
| Human FH | Sense 5'- CCGCTGAAGTAAACCAGGATTATG-3' | 126 |
| | Anti-sense 5'- ATCCAGTCTGCCATACCACGAG-3' | |
| Human MDH2 | Sense 5'- CTGGACATCGTCAGAGCCAACA-3' | 106 |
| | Anti-sense 5'- GGATGATGGTCTTCCCAGCATG-3' | |
